# Ionotropic purinergic receptor 7 (P2X7) channel structure and pharmacology provides insight regarding non-nucleotide agonism

**DOI:** 10.1080/19336950.2024.2355150

**Published:** 2024-05-19

**Authors:** Rua’a Al-Aqtash, Daniel M. Collier

**Affiliations:** Department of Pharmaceutical Sciences, University of Tennessee Health Science Center, Memphis, TN, USA

**Keywords:** P2X7, P2XR, non-nucleotide agonism, extracellular histones

## Abstract

P2X7 is a member of the Ionotropic Purinergic Receptor (P2X) family. The P2X family of receptors is composed of seven (P2X1–7), ligand-gated, nonselective cation channels. Changes in P2X expression have been reported in multiple disease models. P2Xs have large complex extracellular domains that function as receptors for a variety of ligands, including endogenous and synthetic agonists and antagonists. ATP is the canonical agonist. ATP affinity ranges from nanomolar to micromolar for most P2XRs, but P2X7 has uniquely poor ATP affinity. In many physiological settings, it may be difficult to achieve the millimolar extracellular ATP concentrations needed for P2X7 channel activation; however, channel function is implicated in pain sensation, immune cell function, cardiovascular disease, cancer, and osteoporosis. Multiple high-resolution P2X7 structures have been solved in apo-, ATP-, and antagonist-bound states. P2X7 structural data reveal distinct allosteric and orthosteric antagonist-binding sites. Both allosteric and orthosteric P2X7 antagonists are well documented to inhibit ATP-evoked channel current. However, a growing body of evidence supports P2X7 activation by non-nucleotide agonists, including extracellular histone proteins and human cathelicidin-derived peptides (LL-37). Interestingly, P2X7 non-nucleotide agonism is not inhibited by allosteric antagonists, but is inhibited by orthosteric antagonists. Herein, we review P2X7 function with a focus on the efficacy of available pharmacology on P2X7 channel current activation by non-nucleotide agonists in effort to understand agonist/antagonist efficacy, and consider the impact of these data on the current understanding of P2X7 in physiology and disease given these limitations of P2X7-selective antagonists and incomplete knockout mouse models.

## Introduction

Purinergic receptors were first characterized in 1976 [1]. These receptors are classified into two families: P1 adenosine and P2 nucleotide [2]. Within the P2 nucleotide receptor family, there are two distinct subgroups, P2Y metabotropic receptors and P2X ion channels. The P2X family consists of seven genes (p2rx1–7), with proteins designated as P2X1–7. Since their discovery, P2Xs have been implicated in a variety of physiologic and pathologic roles including pain sensation, bone formation, cardiovascular function, and immune system function [3–6].

**Figure 1.**
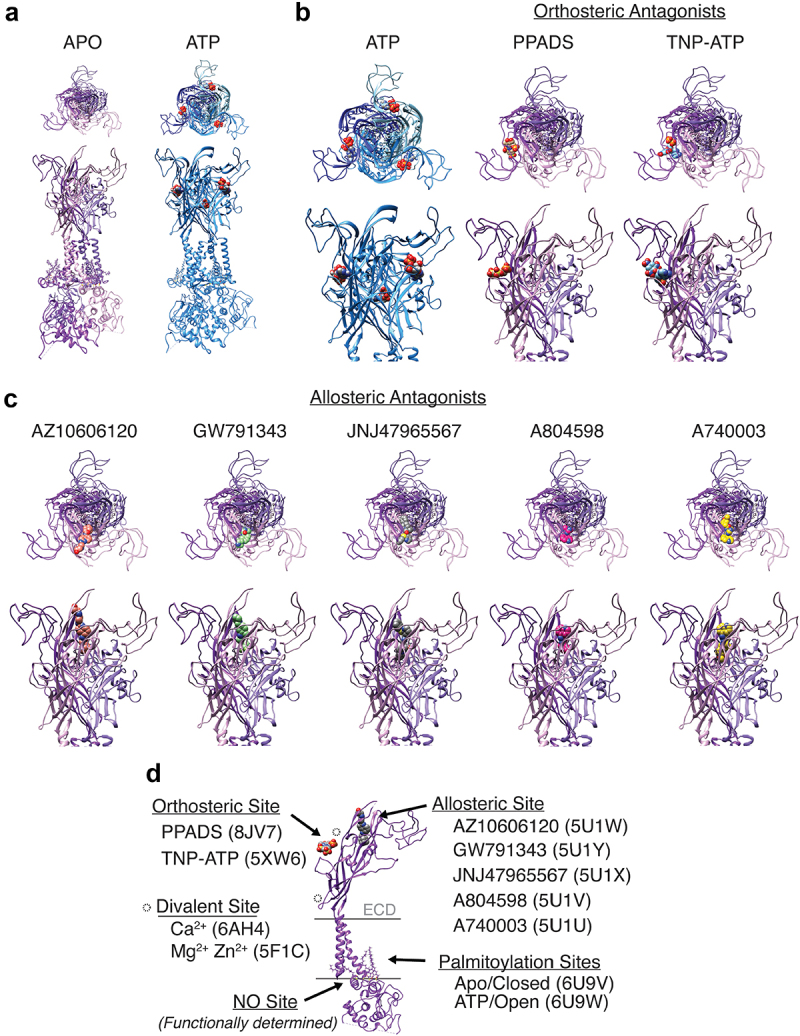
Functionally important binding sites aligned on full-length P2X7 structure. (a) full-length structures of rat P2X7 in the putative closed (Apo, 6U9V) and open (ATP, 6U9W) states. (b,c) top-down (top) and side-on (bottom) views of the three, inter-subunit, ATP binding sites (B, ATP), and single orthosteric binding sites (B) and allosteric binding sites (C). (d) side-on view of a single P2X7 channel subunit (6U9V) to highlight locations of relevant binding sites and functionally important features.

Functional P2X channels are composed of three P2X proteins. P2Xs typically assemble as homomeric channel complexes, although some can form heterotrimeric channel complexes depending on the gene involved [7,8]. Each P2X channel subunit consists of two alpha-helical transmembrane domains (TM) connected by a large extracellular receptor domain (ECD), spanning approximately 269 to 288 amino acids in length, depending on the P2X. The ion channel pore is formed by the second transmembrane domain of each subunit [9–11]. All P2Xs have intracellular N-termini ranging from 20 to 45 amino acids in length. More variation was observed in intracellular C-termini. Typical P2X C-termini are 29–87 amino acids, but P2X7 has the longest C-terminus of 240 amino acids [9,10,12]. High-resolution structural data on P2X channels are available for homotrimers P2X3 (human), −4 (zebrafish), and −7 (panda, chicken, and rat). Of these, only the rat P2X7 structure is a full-length channel, whereas other structures have truncated *N*- and C-termini to facilitate crystallization yet remain functional as cation channels [12]. Full-length P2X7 structures were resolved in both putative open (ATP-bound) and closed (apo) states (Figure 1a) [12]. Differences in these structures provide insights into the regions of the channel that bind ATP, orthosteric, and allosteric antagonist and may be important in regulating channel gating.

P2X7 is unique among the P2XRs in several ways. One of the most apparent differences is that the channel has the lowest affinity for ATP by at least one order of magnitude. Herein, we review unique P2X7 properties described in the literature with the objective of understanding P2X7 current activation by non-nucleotide agonists and the efficacy of P2X7-selective antagonists on this novel mechanism of channel activation. We speculate that a better understanding of P2X7 channel pharmacology may help inform the understanding of P2X7 in physiology and disease.

## P2X7 structure and function

The P2X7 extracellular domain is the site of ATP binding and inhibition by allosteric and orthosteric antagonists [13–15] (Figure 1). Figure 1 shows full-length P2X7 in the putative closed (6U9V, Apo) and open (6U9W, ATP-bound) conformations, as resolved by cryoEM [12]. The ATP-binding site is located at the extracellular interface of the two subunits [12]. For P2X7, the significantly lower affinity for ATP is attributed to the narrowing of the binding domain entrance due to high flexibility in ECD, making it less solvent-exposed [12]. The extracellular domain may also function as a receptor for non-nucleotide agonists. This concept is discussed further in “P2X7 Channel Pharmacology.”

The ion channel pore is composed of a second transmembrane domain of each of the three subunits. In the rat P2X7 structure, the pore radius in the open state is minimally around 2.5 Å with a maximum of 5 Å, compared with 0.1 Å in the closed state [12]. The P2X7 pore dilation hypothesis, which states that the channel transitions from a typical cation conductance state that passes mono- and divalent cations to a second conductance state capable of passing large cations such as NMDG and cationic nuclear dyes, remains unsettled. Evidence of P2X7 direct macromolecule conductance exists, but multiple conductance states have not been observed at the single-channel level [16,17]. The accumulation of macromolecules with prolonged activation may be driven by an increase in the channel open probability (P_o_) rather than by a fundamental change in single-channel conductance [18,19]. This model proposes that macromolecular conductance occurs in a typical open state at a very low permeability ratio. Increasing P2X7 P_o_ sufficiently, and/or for a sufficiently long duration, provides sufficient macromolecule conductance over time to achieve detectable intracellular accumulation. In support of this model, P2X7 is necessary and sufficient for dye uptake when reconstituted in liposomes [20]. This may be the foundation of the reversible permeabilization technique that relies on the activation of P2X7 channel dye uptake after prolonged activation in low Ca^2+^ solutions, as removing extracellular Ca^2+^ increases P2X7 activity [21,22]. However, P2X7 may also activate or recruit macropore-forming channels or hemi channels in biological settings (mechanisms reviewed in [19]). P2X7-mediated dye uptake has been reported to involve pannexin hemi channels and TMEM scramblase activity [18,23,24].

Intracellular *N*- and C-termini are involved in the signaling and regulation of P2X activity. The P2X7 cytoplasmic N-termini and C-termini have been demonstrated to regulate Ca^2+^ conductance [25], kinase binding [26], and receptor desensitization [12]. P2X7 is unique among the P2XRs because it does not desensitize to continued agonist exposure. Desensitization has been shown to be regulated by a juxtamembrane cysteine-rich region that forms a receptor anchor to the plasma membrane through palmitoylation. Mutation of these residues causes P2X7 ATP desensitization [12]. Some palmitoylation residues have also been demonstrated to facilitate P2X7 inhibition by nitric oxide (NO) [27]. The P2X7 intracellular domain contains unique Zn^2+^ and GDP-binding sites, which are thought to be important for intracellular signaling [12]. Disruption of signaling through C-term may underlie the phenotypes reported using the global incomplete knockout model, P2X7 Δ506–532 (Jax #005576; discussed further in “P2X7 knockout mouse models”).

## P2X7 pharmacology

### P2X7 agonism by ATP and ATP derivatives

Extracellular ATP is the canonical agonist of all P2Xs. Despite the highly conserved ATP-binding site in all P2Xs, the seven channels differ widely in their sensitivity to ATP. ATP sensitivity ranges from nanomolar for P2X1 and P2X3, to low micromolar for P2X2 and P2X4, and hundreds of micromolar for P2X7 (with slight variations depending on the species source). P2X activation and desensitization kinetics varies in a similar manner. P2X1 and P2X3 undergo rapid desensitization within milliseconds, whereas P2X2 and P2X4 exhibit slower responses, taking seconds to desensitize. P2X7 again stands out, as it does not desensitize continued ATP exposure [12,28–30]. ATP derivatives, such as BzATP, are potent activators of P2X7, with activation occurring in the micromolar range (Table 1). However, BzATP cannot be regarded as a selective P2X7 agonist. BzATP also activates P2X1–4, although with a similar EC_50_ to that of ATP [72–75].

**Table 1.** P2X7 agonists, antagonists, and modulators: effects on function and/or expression.

Modulation	Ligand/Stimuli	P2X7 investigation Techniques					
		Binding assay	Current recording	Ca2^+^ influx	Dye uptake	IL-1β release	P2X7 expression
Agonist	ATP		EC_50_ = 780 µM Xenopus oocytes [31]	pEC_50_ = 4.13 1321N1 [32]	pEC_50_ = 4.94 1321N1 [32]		
	BzATP		EC_50_ = 52 µM Xenopus oocytes [31]	pEC_50_ = 5.33 1321N1 [32]	pEC_50_ = 6.18 1321N1 [32]		
	Histone		At >3 µg/ml Xenopus oocytes [33]				
	LL-37			At 5 µM HEK293 [34]	At 5 µM HEK293 [34]	At 10 µM monocytes [35]	
	HNP-1	- GST pull-down assay - Antibody assay: Co-localization on HEK-293 [36]			At 50 µg/ml HNP-1 [36]	At 50 µg/ml HNP-1 [36]	
	hβD2				At 20 μg/mL HNP-1 [37]	AT 20 μg/mL HNP-1 [37]	
	SAA					At 30 μg/mL Macrophage [38]	
Antagonists	A438079	Ki = 68 nM Displacement of [3 H]-A-804598 [39]	IC_50_ = 493 nM HEK293 [40]	IC_50_ = 123 nM HEK293 [41]	IC_50_ = 300 nM HEk293 [42]	IC_50_ = 150 nM THP1 [39]	
	A740003	Ki = 130 nM Displacement of [3 H]-A-804598 [39]		IC_50_ = 39.8 HEK293 [43]	IC_50_ = 92 nM THP1 [44]	IC_50_ = 156 nM THP1 [44]	
	A804598	Ki = 8.9 nM [3 H]-A-804598 [39]		IC_50_ = 32 nM HEK293 [39]	IC_50_ = 93 nM THP1 [45]	IC_50_ = 9 nM THP1 [45]	
	A839977			IC_50_ = 20 nM 1321N1 [46]	IC_50_ = 6.61 nM THP1 [46]	IC_50_ = 20 nM THP1 [46]	
	AZ10606120	Kd = 1.4 nM [^3^H]-AZ10606120 [47]	IC_50_ = 10 nM Xenopus oocytes [48]		IC_50_ = 10 nM THP1 [49]		
	AZ11645373		IC_50_ = 31 nM HEK293 [40]		IC_50_ = 19.9 nM THP1 [49]	IC_50_ = 90 nM THP1 [50]	
	AZ11657312				IC_50_ = 474 nM HEK293 [51]		
	GSK1370319A	Ki = 176 nM Displacement of [3 H]-A-804598 [51]				IC_50_ = 3.2 nM Glial cell [52]	
	GW791343				pIC_50_ = 7.2 HEK293 [53]		
	ITH15004				IC_50_ = 9000 nM HEK293 [54]		
	JNJ-47965567	Ki = 12.59 nM Displacement of [3 H]-A804598 [55]		IC_50_ = 35 nM HEK293 [56]	IC_50_ = 15.85 nM HEK293 [56]	IC_50_ = 25.12 nM PBMC [55]	
	Brilliant blue G		IC_50_ = 10 nM HEK293 [57]	IC_50_ >10000 nM HEK293 [39]	pIC_50_ = 5.71 1321N1 [32]	IC_50_ = 700 nM THP1 [39]	
	KN62			pIC_50_ = 4.97 1321N1 [32]	IC_50_ = 175 nM HEK293 [58]	IC_50_ = 12.59 nM THP1 [59]	
	Lu AF27139	Ki = 55 nM Displacement of [3 H]-A-804598 [39]		IC_50_ = 55 nM HEK293 [39]		IC_50_ = 38 nM THP1 [39]	
Expression	oxLDL						Increase [60]
	High glucose						Increase [61]
	Shear stress						Increase [62]
	Hypoxia						Increase [63,64] Decrease [65]
	Cell death, and aging						Increase [66,67]
	Physical exercise						Decrease [68]
	Interleukins, cytokines, TNF-alpha and LPS						Increase [69,70]
	Aβs						Increase [71]

### Endogenous stimuli that alter P2X7 expression level

P2X7 is expressed in a diverse array of cells including immune cells, neurons, glial cells, bone cells, and endothelial cells [76]. Upregulation of P2X7 expression has been observed in various pathologies, including autoimmune diseases, diabetes, hypertension, kidney disease, retinal degradation, cancer, Alzheimer’s, and other CNS-related diseases [77]. These alterations in P2X7 expression are attributed to various endogenous stimuli, encompassing mechanical, metabolic, and inflammatory factors, which modulate P2X7 signaling to elicit a diverse array of cellular responses. Mechanical stimuli, such as shear stress experienced at atheroprone sites, have been documented to upregulate P2X7 expression levels. This augmentation correlates with IL-8 secretion and p38 phosphorylation [62]. Metabolic cues, elevated glucose levels [78–81] and oxidized low-density lipoprotein (oxLDL) [60,82], further contribute to the upregulation of P2X7R expression. This is thought to exacerbate P2X7 mediated inflammatory responses and endothelial dysfunction. Amyloid-β (Aβ) peptides, which play a notable role in Alzheimer’s disease progression by inducing neuroinflammation, increase P2X7 expression [71,83]. A summary of endogenous stimuli and their impact on P2X7 expression levels is presented in Table 1. Upregulation of P2X7 expression may stimulate downstream signaling pathways or increase the channels depolarizing contribution to membrane potential.

### Extracellular non-nucleotide agonists

P2X7 has poor ATP affinity relative to other P2XRs. This raises questions on the physiological mechanisms of P2X7 activation, as it may be difficult to reach the near-millimolar extracellular ATP concentrations needed. The pioneering work by Elssner and colleagues demonstrated that the P2X7 channel can be activated by the non-nucleotide agonist LL-37, a human antimicrobial peptide (AMP) from the cathelicidin family [84]. They observed that human monocytes expressing P2X7 channels released IL-1β and exhibited dye uptake upon exposure to LL-37, independent of ATP release [84]. Subsequent studies by other research groups have further investigated LL-37 effects, confirming its ability to induce P2X7 channel-mediated Ca^2+^ influx, dye permeation, IL-1β release, and enhance P2X7 sensitivity to ATP [34,85,86]. There is currently a lack of data on LL-37 activation of P2X7 channel current. Further work is needed to understand the mechanism of LL-37 action on P2X7.

A variety of non-nucleotide P2X7 ligands have been reported (Table 1). Human neutrophil peptide 1 (HNP-1) from the human AMP α-defensins family has been shown to directly bind with the P2X7 receptor (using glutathione S-transferase (GST) pull-down assay) and stimulate dye uptake and IL-1β release [36,87]. Human beta-defensin 2 (hβD2) from the human AMP β-defensins family enhances IL-1β production through P2X7-mediated NLRP3 expression in macrophages, although the direct interaction with the channel remains unclear [37]. High-density apolipoproteins, such as acute-phase reactant serum amyloid A (SAA), have been demonstrated to mediate inflammasome activation through interaction with P2X7R in LPS unprimed macrophages, independent of ATP release [38,88,89]. Di Virgilio and colleagues provide a detailed review on non-nucleotide agonism and pore formation [90]. Since this work, extracellular histone proteins have been identified as novel agonists of P2X7 ionic current (discussed in detail below in “Extracellular histone proteins as P2X7 agonists”) [33].

Non-nucleotide agonists action on P2X7 have predominately been assessed using dye- or fluorescence-based flux assays and downstream reporters, such as measurement of IL-1β release. Many questions remain regarding the mechanisms underlying non-nucleotide agonism. Do these ligands function as true agonists independent of ATP or do they act as positive modulators that enhance P2X7 sensitivity to ATP or other stimuli? If they enhance their sensitivity, how do they achieve this? Is this through increased levels of circulating ATP, higher receptor expression, or a combination of both? Thus, there is a strong need for more direct functional assays, such as current recording, labeled ligand studies, and co-crystallization, to elucidate binding and activation mechanisms.

### Extracellular histone proteins as P2X7 agonists

Histone proteins have been recognized as intranuclear DNA-binding proteins that have been crucially involved in gene regulation for over a century, with their discovery credited to Albrecht Kossel in 1884 [91]. These proteins are characterized by their alkaline nature and high degree of conservation across species [92]. Within the nucleus, post-translational modifications of histones are pivotal for governing chromatin structure and controlling gene expression [93,94]. However, histone proteins are also found and have functions in the extracellular space.

Elevated levels of circulating histone proteins have been reported in various diseases, including infectious conditions such as sepsis and COVID-19, as well as sterile inflammation-related diseases, including traumatic/ischemic injury, cancer, and autoimmune diseases [95–100]. Studies have reported increased levels of autoantibodies targeting histone proteins in individuals with autoimmune disorders such as systemic lupus erythematosus (SLE) [101,102]. Histone proteins can be released from the nucleus into the circulation through passive and active mechanisms. These mechanisms encompass any type of cell death, including apoptosis, necrosis, and NETosis, as well as active release from living cells through exosomes [103–106]. Bell et al. proposed that histone proteins act as immunogenic triggers for B cells [107]. More recently, the release of histones has been associated with endothelial cell injury and increased inflammatory signaling, resulting in multiple organ damage [98,108]. However, the molecular mechanisms underlying histone signaling are not fully understood.

The literature presents two primary hypotheses to explain histone action. First, extracellular histones function as antimicrobial peptides/proteins (AMPS) and directly interact with plasma membrane phospholipids, leading to membrane disruption and Ca^2+^. Second, extracellular histones serve as damage-associated molecular pattern molecules (DAMPs) that can be recognized by host pattern recognition receptors, resulting in the release of proinflammatory cytokines.

In the first model, histones were thought to be AMPs [109]. AMPs are highly basic because of their high number of Arg and Lys residues. This allows AMPs to bind to lipopolysaccharides of gram-negative bacteria and teichoic acids of gram-positive bacteria. Such interactions result in disruption of the bacterial cell membrane, or histones may even penetrate the bacterial membrane and bind directly with bacterial DNA [110]. However, AMPs lose membrane-disruptive properties in mammalian cells in the serum, suggesting that their host actions rely on other signaling pathways [111]. This leads to the second model, wherein histones are considered DAMPS.

Histone protein levels are normally around 2 microgram/ml in human circulation, but increase by five- to more than fifty-fold after injury and are believed to contribute to systemic vascular dysfunction [97,112,113]. Circulating histone proteins are also elevated through neutrophil recruitment and the release of histone-rich neutrophil extracellular traps (NETs) in the innate immune response [114–116]. It has been shown that the pathogenesis of COVID-19 vascular dysfunction is driven by neutrophil activation, elevated NET formation, and increased circulating DAMPs/AMPs [117–119]. Studies in mammalian cells support the ability of histones to bind to membrane phospholipids and cause membrane disruption [120,121]. Others suggest that histone binds to phospholipid phosphate groups in a manner similar to DNA [112,122]. The extent of histone core and subunit penetration is reported to be positively correlated with increased membrane negative charge and inversely with cholesterol component, except for H2B, which fails to penetrate the tested membranes [123,124]. As negatively charged phospholipids reside in the inner leaflet of most mammalian membranes and have a positive charge at the outer surface [125], the forces or mechanisms that bring basic histone proteins in close contact with the inner membrane are unclear.

Another possibility is that histone proteins exert their effects on the cells through membrane receptors. The full repertoire of receptors for extracellular histone proteins remains unknown. Multiple studies provide evidence of histone activation of Toll-like receptors (TLRs) 2, −4, and −9 and subsequent release of host immune modulators such as TNF-‹, IL-6, IL-1β, IL-18, CXCL9, and CXCL10 (for more details, we recommend the following reviews [98,99,121]). The main caveat of this model is that it does not explain the rapid histone-induced Ca^2+^ influx observed in the vascular endothelium [126]. TLR activation can induce Ca^2+^ release from intracellular stores [121]. Histone-induced Ca^2+^ influx is not dependent on Ca^2+^ release and is still present in TLR4 knockout mice [126]. This suggests that TLRs are not the only receptors that can be activated by circulating histones. Al-Aqtash et al. recently provided evidence for histone proteins as novel P2X7 agonists [33]. Importantly, in this study, P2X7 expression was necessary and sufficient for histone-induced current [33]. These data may explain the histone-mediated effects in P2X7 expressing cells including vascular endothelial cells and circulating immune cells. However, data on histone-evoked P2X7 currents are currently limited to a heterologous expression system due to the limitations of commercially available P2X7 knockout mouse models (discussed in below “P2X7 knockout mouse models”) and limited efficacy of P2X7-selective allosteric antagonists on non-nucleotide-evoked current (discussed in below in “P2X7 antagonists”). New conditional mouse models will be necessary to thoroughly investigate histone-induced P2X7 channel current in native tissue.

### P2X7 antagonists

Multiple P2X-selective and nonselective antagonists are commercially available [127–129]. P2X7 ATP-evoked currents are inhibited by both selective and nonselective antagonists. The binding sites of some of these have been determined through structural and functional studies [11,13,15,130,131]. P2X7-selective antagonists bind to an allosteric binding site at the interface of the two subunits near the crown of the extracellular domain. We used Chimera to align all available allosteric agonist-bound structures to the full-length closed state P2X7 structure to compare binding sites (6U9V, Figure 1) [132]. All the allosteric inhibitors bind to the same region. Figure 1c shows allosteric antagonists aligned with the closed full-length structure. The P2X7 allosteric inhibitors and available co-crystals are summarized in Figure 1d and Tables 1 and 2.

**Table 2.** P2X7-selective and nonselective antagonist co-crystals.

Non-selective P2X inhibitors	Inhibit Histone Current?	P2X7 co-crystal	Ref
Suramin	Yes	NA	[133]
Reactive Blue 2	–	NA	[134]
TNP-ATP (Orthostatic inhibitors)	Yes	5XW6/chicken	[135]
PPNDS (Orthostatic inhibitors)	–	8JV8/Panda	[14]
PPADS (Orthostatic inhibitors)	Yes	8JV7/Panda	[14]
**P2X7-selective inhibitors**			
A438079	No	NA	[136]
A740003	–	5U1U/Panda	[13]
A804598	–	5U2H (ATP bound)/Panda	[13]
A804598	–	5U1V/Panda	[13]
A839977	–	NA	[137]
AZ10606120	No	5U1W/Panda	[13]
AZ11645373	No	*Functionally Mapped	[15,138]
AZ11657312	–	NA	[139]
GSK1370319A	–	NA	[51]
GW791343	No	5U1Y/Panda	[13]
ITH15004	–	NA	[54]
JNJ-47965567	–	5U1X/Panda	[13]
JNJ-47865567	–	NA	[140]
JNJ-42253432	–	NA	[141]
JNJ-54166060	–	NA	[140]
LuAF27139	–	NA	[39]
Brilliant blue G	–	*Functionally Mapped	[15,57]
KN62	No	*Functionally Mapped	[15,142]
**Radio labeled P2X7-selective inhibitors**			
[11C] JNJ-54173717	–	NA	[143]
[3 H] JNJ-54232334	–	NA	[144]
[11C] GSK1482160	–	NA	[145]

P2X7 orthosteric antagonists block channel activity by competing with ATP-binding. High-resolution structures are available for TNP-ATP, PPADS, and PPNDS bound to the P2X7 ECD [14,135]. All antagonists, allosteric or orthosteric, inhibited P2X7 activation by ATP, albeit with varying IC_50_ values. However, only orthosteric antagonists have been shown to inhibit P2X7 activation by non-nucleotide agonists [146,147]. Al-Aqtash et al. showed that P2X7 current activation by histone proteins is not blocked by selective allosteric P2X7 inhibitors [33]. This aligns with previous studies on LL-37, where selective P2X7 antagonists failed to inhibit P2X7-mediated dye uptake [146]. While the physiological significance of this requires further investigation, we speculate that this agonist/antagonist dependence may impact our understanding of P2X7 physiology and interpretation of antagonist efficacy. These findings suggest that histones and LL-37 bind to a distinct site that is unaffected by allosteric antagonists designed to inhibit P2X7 activation by ATP. New structural studies are needed to determine the sites and mechanisms of agonism by non-nucleotide agonists to facilitate the discovery and development of more effective P2X7-selective antagonists.

P2X7 has been actively targeted in clinical trials because of its important role in modulating the innate and adaptive immune systems without suppressing host immunity [148]. Multiple P2X7 selective antagonists have been developed and tested for multiple inflammatory diseases, with autoimmune and neurodegenerative diseases being at the top of the list [149,150]. Table 3 lists selective P2X7 antagonists in both preclinical and clinical trials and their suggested use. AstraZeneca and Pfizer initially investigated in clinical trials to evaluate P2X7 antagonists for autoimmune disorders, such as rheumatoid arthritis, osteoarthritis, and Crohn’s disease. Meanwhile, GSK and Janssen explored the effects of P2X7 antagonists on neuroinflammatory and central nervous system (CNS) diseases, making notable progress in the development of CNS-permeable variants. However, none of the developed highly selective P2X7 antagonists have secured a place in the market and have failed to demonstrate efficacy in advanced (Phase II/III) clinical trials, despite their strong safety profile (Table 3). This lack of success with small-molecule inhibitors is surprising, given the breadth of physiological roles [158–160]. The only clinically successful P2X7 focused treatment reported to date relies on a CAR T-cell approach by Biosceptre, which targets a nonfunctional variant of the P2X7 receptor (nfP2X7). This variant is highly expressed in multiple cancer types but not in healthy cells [161]. Additionally, nfP2X7 antibodies do not recognize functional P2XR7variants, which allows treatment to be selective for tumor cells but minimizes the likelihood of any use to inhibit P2X7 function [156].

**Table 3.** P2X7 inhibitors in clinical trials.

Compound	Indication	Phase	Results	Company	NCT Number	Ref
JNJ-54175446	Major depression	II	NA	Janssen Pharmaceuticals	NCT04116606	[151]
JNJ-55308942	Bipolar depression	II	NA	Janssen Pharmaceuticals	NCT05328297	[152]
PTM-001	Hidradenitis Suppurativa	II	NA	Phoenicis Therapeutics	NCT05020730	[152]
CE-224,535	Rheumatoid arthritis	II	No effect	Pfizer	NCT00628095	[153]
AZD-9056	Rheumatoid Arthritis	II	No effect	AstraZeneca	NCT00520572	[154]
GSK1482160	Inflammatory pain	I	No effect	GlaxoSmithKline	NCT00849134	[155]
BSCT (Anti-nf-P2X7)	Basal Cell Carcinoma	I	Reduced BCC size	Biosceptre	NCT02587819	[156]
SGM-1019	Non-alcoholic steatohepatitis	II	Unfavorable risk/benefit	Second Genome	NCT03676231	[157]
[18F] JNJ-64413739	Glioblastoma/Diagnostic test: PET imaging	NA	NA	Janssen Pharmaceuticals	NCT05753995	[156]

### P2X7R endogenous inhibitors

P2X7 receptors are inactive under physiological conditions. This is because of the high levels of extracellular ATP required for activation (mM range) as well as receptor inhibition by divalent cations, including zinc, magnesium, calcium, and copper [162]. Divalent cations chelate the free acid form of ATP (ATP^4-^) [163], directly interact with the receptor’s extracellular domain, and allosterically affect agonist binding [164]. The potency of divalent cation inhibition varies among different cations and across species; therefore, caution is necessary when extrapolating animal data to humans [165]. Similarly, the lipid component of the plasma membrane significantly influences P2X7 function. Studies have strongly supported the inhibition of P2X7 through direct cholesterol binding to the transmembrane domain [20,166,167]. Other data suggest that P2X7 activity is inhibited through the activation of independent endogenous pathways. Richter et al. reported that nicotinic receptor activation inhibits the inotropic function of P2X7R through the activation of eNOS and production of NO, which inhibits P2X7 function through cysteine 377 at the cysteine-rich C-terminus. The authors proposed that this serves as an endogenous anti-inflammatory mechanism [27].

### P2X7 knockout mouse models

Currently, there are three global P2X7 knockout (KO) mouse models. GSK and Pfizer are developed models that are available and have been used extensively in the literature to assess P2X7 enrollment in different disease studies. GSK was the pioneer in introducing a global P2X7 KO model by deletion of exon 1 through insertion of a LacZ-neomycin cassette into the 5’ end of exon 1 [168]. Although these animals lack the fully functional P2X7 A isoform, P2X7R K is another functional splice variant that escapes deletion and is encoded by an alternative exon 1 in mice. This isoform has a higher affinity for ATP and is highly expressed in T lymphocytes [169]. Pfizer developed a global P2X7 knockout by targeting the C-terminal domain (P2X7 Δ506–532, “P2X7 Δ C-term,” Jax #005576) by neomycin cassette insertion into exon 13 [170]. This results in truncated P2X7 A and K isoforms, but there are still other splice variants that escape this deletion and are still functional in these animals – P2X7 13B, and 13C [171]. Lexicon Genetics substituted exons 2 and 3 with the LacZ-neomycin cassette, which disrupts the development of any P2X7 splice variant [172]. However, these mouse strains are not widely used in literature. Thus, there is a need for new conditional knockout models to overcome the limitations of global and/or incomplete P2X7 knockout. For further details about P2X7 animal models and genetics, we recommend the following reviews [173–175]

## Future needs and therapeutic implications

Surprisingly, given the broad expression profile and numerous roles of P2X7 in physiology and disease, clinical trials using selective P2X7 antagonists have been ineffective. Current limitations in pharmacology and incomplete and/or global KO mouse models leave much to be resolved regarding the roles of P2X7 and its effective channel modulation in physiology and disease. Differences in the efficacy of orthosteric and allosteric P2X7 antagonists may play a role in the lack of clinical efficacy observed. While orthosteric antagonists inhibit P2X7 activation by ATP and non-nucleotide agonists (histone proteins, LL37), P2X7-selective allosteric antagonists inhibit only channel activation by ATP. Orthosteric antagonists are not ideal clinical solutions, because they lack P2X7 selectivity and thus could cause off-target effects by inhibiting other P2Xs. These pharmacological differences suggest that the mechanisms by which ATP and non-nucleotide agonists gate P2X7 channels differ. To better understand the role of P2X7 channel function in physiology and disease, new pharmacology that is both receptor-specific and effective against non-nucleotide agonism and new conditional knockout mouse models are needed. For example, CAR-T cell approaches targeting nfP2X7 have shown some clinical success [161]. Translation of this to functional P2X7 channels may provide a means of decreasing P2X7 activity while bypassing the efficacy and selectivity issues of allosteric and orthosteric P2X7 antagonists. As we learn more about differences in P2X7 splice variant expression at the single-cell level, CAR-T cell therapy approaches may also be able to provide tissue-specific regulation of functional P2X7. We speculate that new antagonists and a better understanding of tissue-specific function and expression may improve the translation from basic science to therapeutic intervention.

## Data Availability

Data sharing is not applicable to this article as no new data were created in this study.
